# TAG pathway engineering via GPAT2 concurrently potentiates abiotic stress tolerance and oleaginicity in *Phaeodactylum tricornutum*

**DOI:** 10.1186/s13068-020-01799-5

**Published:** 2020-09-14

**Authors:** Xiang Wang, Si-Fen Liu, Ruo-Yu Li, Wei-Dong Yang, Jie-Sheng Liu, Carol Sze Ki Lin, Srinivasan Balamurugan, Hong-Ye Li

**Affiliations:** 1 Key Laboratory of Eutrophication and Red Tide Prevention of Guangdong Higher Education Institutes, College of Life Science, Jinan University, Guangzhou, 510632 China; 2 School of Energy and Environment, City University of Hong Kong, Tat Chee Avenue, Kowloon, Hong Kong, China; 3 Department of Biotechnology, Bharathidasan University, Tiruchirappalli, 620024 India

**Keywords:** Abiotic stress tolerance, Diatom, Glycerol-3-phosphate acyltransferase, Lipid hyperaccumulation, Lipid remodeling

## Abstract

**Background:**

Despite the great potential of marine diatoms in biofuel sector, commercially viable biofuel production from native diatom strain is impractical. Targeted engineering of TAG pathway represents a promising approach; however, recruitment of potential candidate has been regarded as critical. Here, we identified a glycerol-3-phosphate acyltransferase 2 (GPAT2) isoform and overexpressed in *Phaeodactylum tricornutum*.

**Results:**

GPAT2 overexpression did not impair growth and photosynthesis. GPAT2 overexpression reduced carbohydrates and protein content, however, lipid content were significantly increased. Specifically, TAG content was notably increased by 2.9-fold than phospho- and glyco-lipids. GPAT2 overexpression elicited the push-and-pull strategy by increasing the abundance of substrates for the subsequent metabolic enzymes, thereby increased the expression of *LPAAT* and *DGAT*. Besides, GPAT2-mediated lipid overproduction coordinated the expression of NADPH biosynthetic genes. GPAT2 altered the fatty acid profile in TAGs with C16:0 as the predominant fatty acid moieties. We further investigated the impact of GPAT2 on conferring abiotic stress, which exhibited enhanced tolerance to hyposaline (70%) and chilling (10 ºC) conditions via altered fatty acid saturation level.

**Conclusions:**

Collectively, our results exemplified the critical role of GPAT2 in hyperaccumulating TAGs with altered fatty acid profile, which in turn uphold resistance to abiotic stress conditions.

## Background

Photosynthetic microalgae harbor the biosynthetic machinery to synthesize high-value biochemicals using carbon dioxide as the substrate, thereby emerging as the sustainable microbial cell factories and potentially offering promising solutions for contemporary commercial and environmental issues [1]. Particularly, triacylglycerides (TAGs) from oleaginous microalgae have been regarded as the most preferable feedstock for the production of biodiesel and a range of valuable oleochemicals [2]. Diatoms are the predominant phytoplankton species that are distantly related to vascular plants which acquired their photosynthetic machinery through endosymbiosis of red algae, meanwhile, diatom contribute almost 25% of total oxygen generation on the Earth [3]. Among, pennate diatom *Phaeodactylum tricornutum* has been considered the robust model organism and possibly the well-studied of all diatoms till date, owing to their various fascinating inherent characteristics such as promising oleaginicity, rapid growth rate, potential to grow in a wide range of medium, capability to accumulate various biocomponents, availability of sequenced genome and optimized genetic toolbox, and capability to exhibit comparable growth in outdoor large-scale cultivation systems [4, 5]. Owing to these characteristics, *P. tricornutum* is presently of great interest as potential and substantial alternatives for petrofuels and other commodity components production. *P. tricornutum* naturally accumulates considerable content of lipids, particularly when the cells were subjected to stress conditions but at the cost of cellular growth and overall productivity, thereby provided the impetus to genetically improve the strain to meet the commercial challenges.

Targeted engineering of lipogenic pathway has exemplified the potential strategy to increase the lipid content in transgenic strain without impairing cellular physiological characteristics. However, previous research attempts have yielded mixed results as the lipogenesis is being governed by various layers of regulatory components [6–8]. Among the metabolic targets for lipogenic engineering, precise perturbation of TAG pathway has been regarded as the most straightforward approach to enhance algal lipid content. Burgeoning investigations and research efforts have offered us an in-depth understanding of the intricate biosynthetic machinery of TAGs in *P. tricornutum* at genetic level. Recent reports have demonstrated the occurrence of plastidial located TAG biosynthetic pathway in addition to the conventional endoplasmic reticulum localized Kennedy pathway, which advanced our understanding of TAG biogenesis along with fatty acid acylation pattern of the accumulated TAGs [6, 7, 9]. However, there is still much room for further improvement of microalgal lipogenesis along with the engineered fatty acid profile.

Glycerol-3-phosphate acyltransferase (GPAT; E.C. 2.3.1.15) is the first rate-limiting enzyme that catalyzes the acylation of the glycerol backbone to form lysophosphatidic acid (LPA) from glycerol-3-phosphate (G-3-P), which is subsequently acylated into TAGs via TAG pathway. It has been identified that *P. tricornutum* genome possesses 5 GPAT isoforms, whereas mammals have 4 isoforms of GPAT, however, unlike the characterization of DGATs, the mechanistic role of GPATs on governing lipid accumulation is yet to be elucidated, particularly in pennate diatoms despite their economic and biological importance. It has been shown that GPAT of *Paeonia lactiflora* plays a pivotal role in conferring cold stress resistance by possibly altering the fatty acid profile, which in turn altered the membrane fluidity [10]. Similarly, heterologous expression of GPAT from *Suaeda salsa* enhanced the germination rate and root length in transgenic *Arabidopsis* under salt stress probably by maintaining the unsaturated fatty acids of phosphatidylglycerol, whereas the control plants were severely affected [11]. However, the mechanistic role of GPAT is limited up to lipogenesis and there are very few reports available on its role in microalgae other than lipogenesis. In this study, we identified the GPAT2 in *P. tricornutum* which reinforced its lipogenic role in diatom and interestingly, our results uncovered the crucial role of GPAT in conferring resistance to chilling and hyposaline stress conditions.

## Results

### In silico analyses of GPAT2 sequence

GPAT2 sequence (XP_002184838.1) was identified and the conserved domains of the identified GPAT2 were predicted by bioinformatic tools. The identified GPAT2 gene encodes a protein comprising 435 amino acid resides. As shown in Additional file 1: Fig. S1A, the GPAT2 protein contains an LPLAT super family between 193 and 419 amino acids in the N-terminal and also a transmembrane domain (12–30 amino acids) in its N-terminal portion, demonstrating that GPAT2 could be the potential membrane protein. In order to uncover the phylogenetic relation of the identified GPAT2, we constructed the phylogenetic tree using MEGA. Interestingly, phylogenetic tree revealed that the identified GPAT2 closely related with the three GPAT isoforms of *Brassica napus* and they belonged to the same clade, while at the same exhibited highly divergent from other GPATs of *P. tricornutum* which are distantly related (Additional file 1: Fig. S1B). In silico prediction of the localization of GPAT2 showed that the identified GPAT2 might be targeted to chloroplast. These results suggest that GPAT2 was well conserved during evolutionary development, having close relationships with GPAT isoforms from *B. napus* and plastidial ATS1 from *Arabidopsis*, while remaining far from other two homologous GPAT isoforms.

### Molecular validation of transgenic strains

**Fig. 1:**
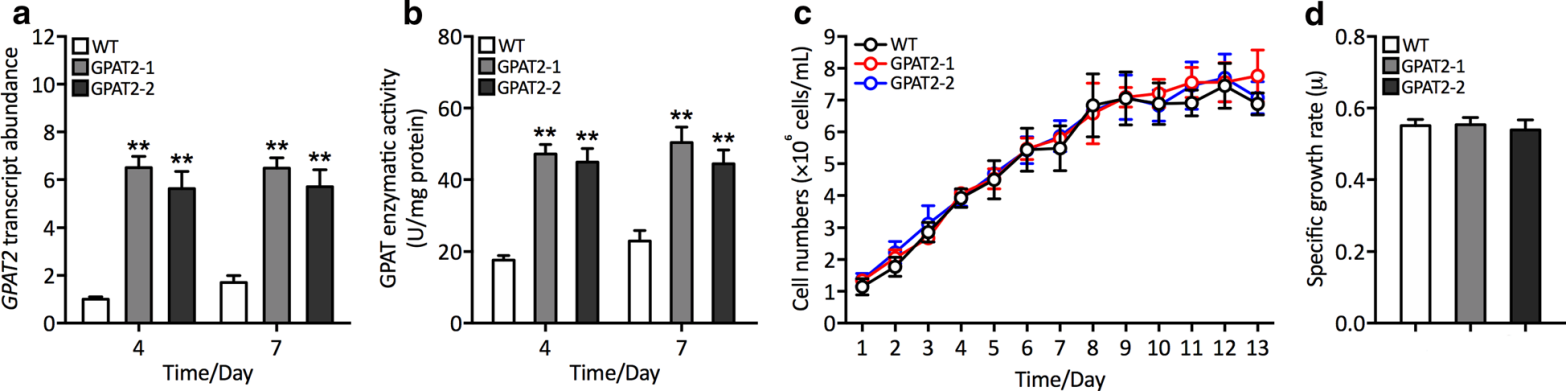
Molecular and physiological characterization of engineered cells overexpressing GPAT2. **a** Relative transcript abundance of GPAT2 as determined by using qPCR using β-actin as the internal reference gene. **b** Determination of enzymatic activity during 4th and 7th day of cultivation under optimal conditions. **c** Growth rate of engineered and WT strains as determined by direct cell count method. **d** Specific growth rate (*µ*) of engineered and WT strains. Significant difference is indicated at the *p* < 0.05 (*) or *p* < 0.01 (**) level. Each value represents the mean ± SD (*n* = 3)

To further demonstrate the mechanistic role of the GPAT2 in *P. tricornutum*, we amplified the full-length coding sequence of GPAT2 by PCR and cloned into the expression vector pHY18 and subsequently electroporated into electrocompetent *P. tricornutum* cells. Putative transgenic cells raised from the selection medium containing chloramphenicol were picked and cultured for five cycles of subculture and subjected to molecular characterization. Several independent overexpressing cells with uniform phenotypes were randomly selected and subjected for further analysis. Firstly, we performed the colony genomic PCR and it was carried out using the primers to amplify the introduction of expression cassette into the host cells. PCR analyses revealed the presence of PCR amplicon corresponding to the chloramphenicol acetyltransferase encoding gene only in the transgenic cells, but no such band was detected in wild type (WT), which confirmed that the expression cassette was successfully introduced into the genome of the transgenic cells (data not shown). To determine the relative transcript level of the introduced GPAT2 in transgenic cells, real-time quantitative PCR (qPCR) analysis was performed. As shown in Fig. 1a, qPCR data demonstrated that relative mRNA level of GPAT2 was significantly increased than that of WT during 4th and 7th day of cultivation in both the transgenic cells than that of WT. To further validate the expression of GPAT2 in transgenic cells, we determined the enzymatic activity of GPAT2, which showed that activity of GPAT2 was significantly higher in transgenic cells during 4th and 7th day of cultivation, particularly the observed increment in enzymatic activity was notable during 7th day of cultivation (Fig. 1b). Together, these data demonstrated that the introduced GPAT2 was successfully introduced, transcribed and expressed in the transgenic lines GPAT2-1 and GPAT2-2.

### GPAT2 overexpression did not impair general cellular physiological parameters

Previous studies have shown that attempts to overproduce lipids in microalgae resulted in inconsistent success, where the transgenic cells exhibited impaired growth rate and consequently hindered the commercial application of microalgae for biofuel production, thus, it is of importance to examine the physiological properties of the transgenic cells [12, 13]. To gain more physiological implications of GPAT2 overexpression in transgenic cells, we determined the growth curve by direct cell count method, specific growth rate and various photosynthetic parameters. Growth analyses as determined by direct cell count and specific growth rate revealed that there was no difference observed between transgenic and WT cells (Fig. 1c, d). In order to further uncover the role of GPAT2 overexpression on photosynthesis, we determined the crucial photosynthetic parameters such as Fv/Fm, the maximum quantum yield of photosystem II, chlorophyll *α* content, non-photo chemical quenching and electron transport rate. As shown in Additional file 1: Fig. S2, there was no significant difference observed between WT and transgenic cells in terms of these photosynthetic factors and demonstrated that GPAT2 overexpression did not affect growth and photosynthetic characteristics negatively in transgenic *P. tricornutum*.

### Analysis of primary metabolites corroborated the lipogenic role of GPAT2 likewise its other isoforms

**Fig. 2:**
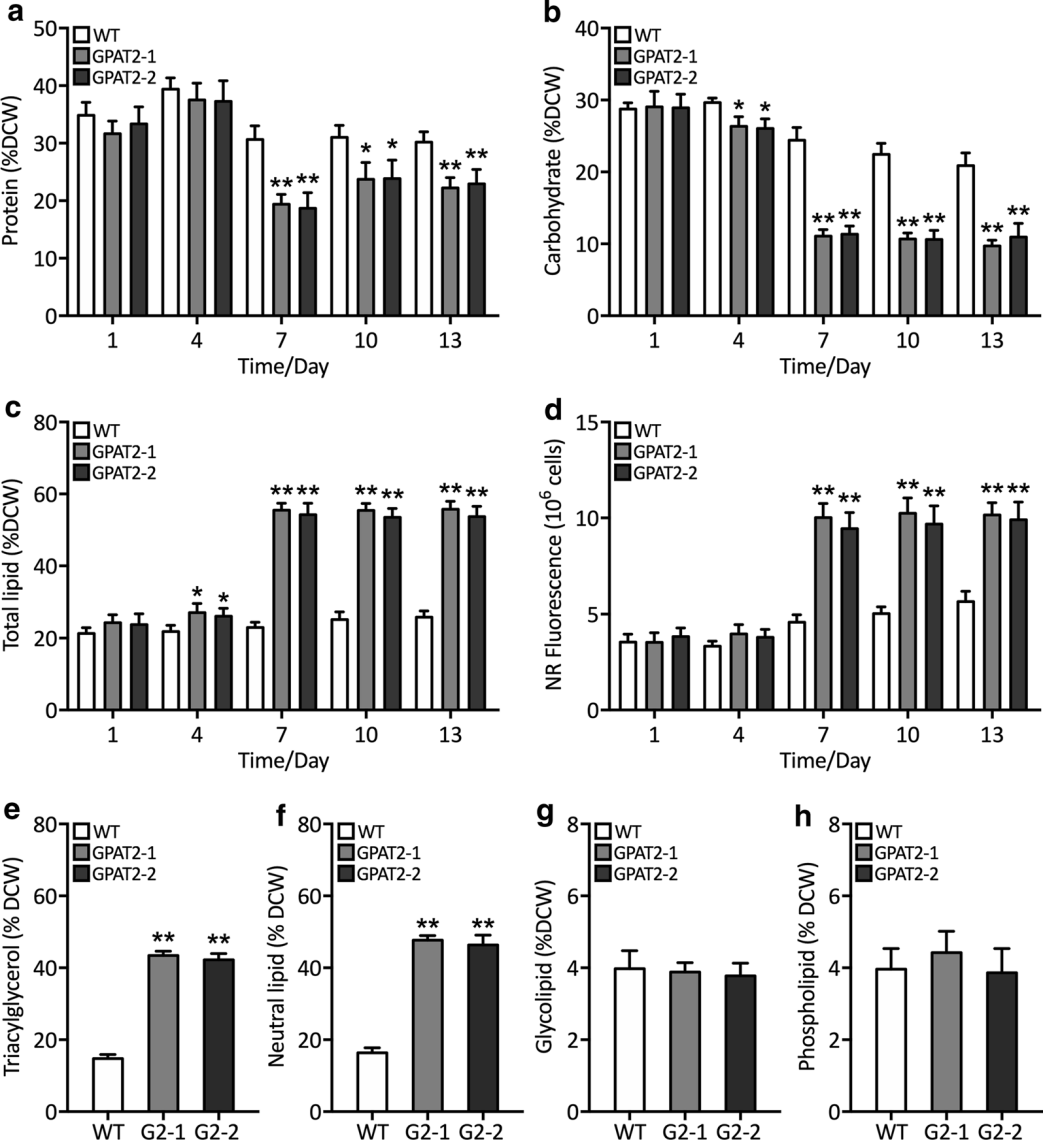
Analysis of primary metabolite and relative neutral lipid content. **a** Determination of total soluble protein content (% DCW). **b** Determination of total carbohydrate content (% DCW). **c** Total lipid content (% DCW) as determined gravimetrically. **d** Relative neutral lipid content as measured by Nile-red fluorometric analysis during growth phase. Determination of **e** TAG content (% DCW), **f** neutral lipid content (% DCW), **g** glycolipid (% DCW), **h** phospholipid (% DCW). Significant difference is indicated at the *p* < 0.05 (*) or *p* < 0.01 (**) level. Each value represents the mean ± SD (*n* = 3)

Previous reports have investigated the impact of GPAT overexpression on elevating lipid content in various systems [14, 15]. Here, we attempted to gain more insights into the mechanistic role of the identified GPAT2 in transgenic *P. tricornutum*. To this end, we determined the primary metabolite content such as total carbohydrate, protein and lipid content in transgenic cells. As shown in Fig. 2a–d, there was no significant difference in terms of protein, lipid and carbohydrate content till 7th day of growth curve. However, total protein and carbohydrate content were found to be significantly decreased in transgenic cells after 7th day of cultivation. On the other hand, total lipid content was determined by both qualitative fluorometric and quantitative gravimetric method, which showed that lipid content was found to be significantly increased in both the transgenic strains than that of WT. These results showed that overexpression of GPAT2 increased the TAG biosynthetic reaction, which redirected the carbon metabolic precursors from carbohydrate biosynthetic pathway towards lipid biosynthetic pathway, which is in line with the previous reports [9].

### Lipid fractionation of transgenics uncovers the role of GPAT2

To further characterize the mechanistic role of GPAT2 on lipid biosynthesis, we fractionated the total lipid content by thin-layer chromatography and solid-phase extraction (SPE). As shown in Fig. 2e, f, GPAT2 overexpression resulted in significant elevation of neutral lipids and TAGs than that of the WT cells. On the other hand, there was no notable difference observed in terms of phospholipid and glycolipid between transgenics and WT (Fig. 2g, h). These data corroborated the crucial of GPAT2 on governing the TAG biosynthetic pathway in *P. tricornutum*.

### Analyses of key TAG biosynthetic genes expression pattern uncovered that GPAT2 overexpression elicited the push-and-pull strategy that underpins TAG overproduction

**Fig. 3:**
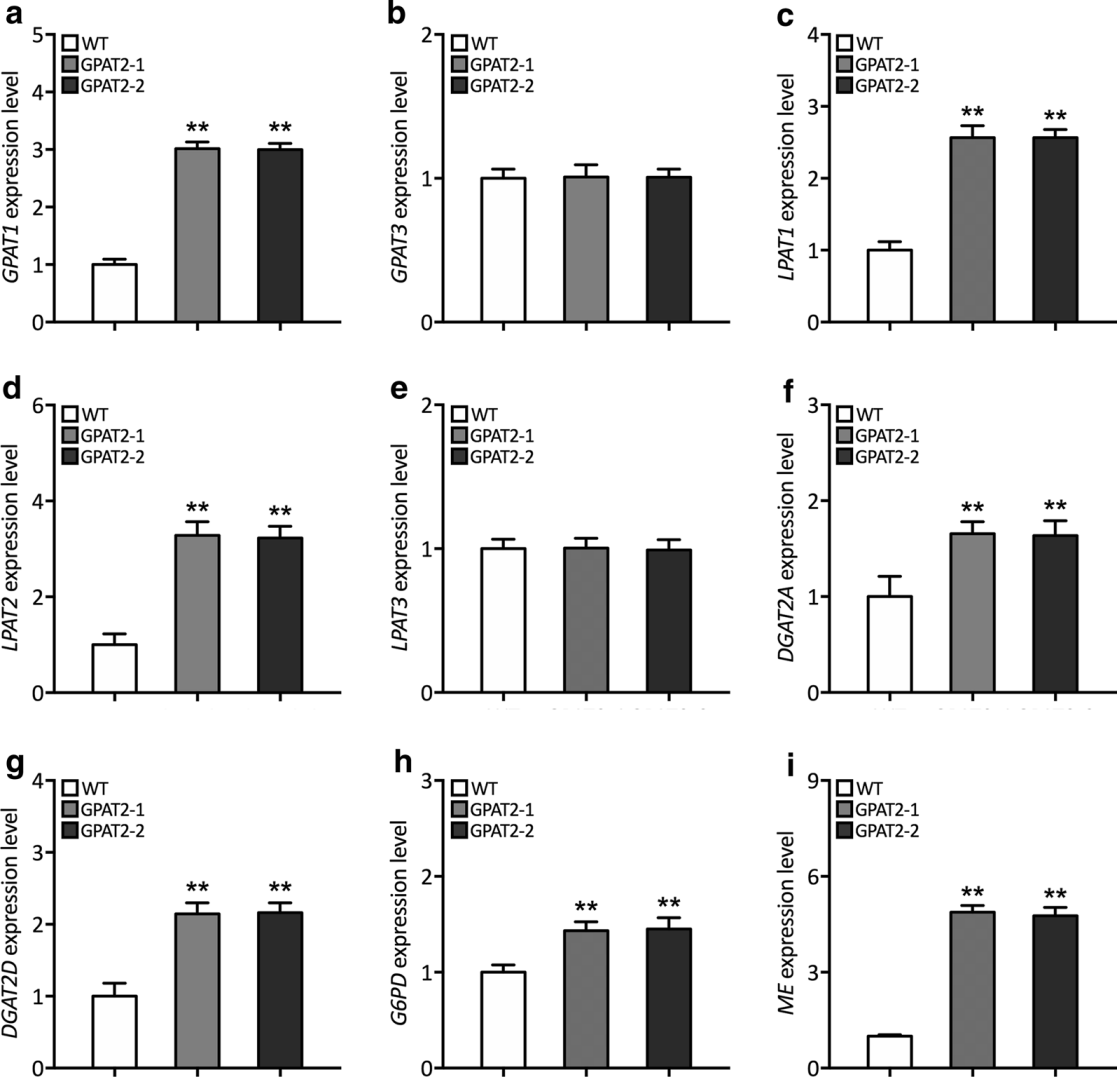
Analysis of expression pattern of key TAG biosynthetic genes. Relative transcript abundance of **a** *GPAT1*, **b** *GPAT3*, **c** *LPAT1*, **d** *LPAT2*, **e** *LPAT3*, **f** *DGAT2A*, **g** *DGAT2D*, **h** *G6PD*, and **i** *ME* as determined by qPCR using β-actin as the internal reference gene. Significant difference is indicated at the *p* < 0.05 (*) or *p* < 0.01 (**) level. Each value represents the mean ± SD (*n* = 3)

Given the significant increment of TAG overproduction in transgenic cells, we further determined the expression level of key TAG lipogenic genes such as *GPAT*, *LPAT*, *DGAT*, *ME* and *G6PD* in order to gain insight into the mechanistic role of GPAT2. We firstly examined the expression pattern of GPAT isoforms, and found the chloroplast-localized GPAT1 was simultaneously upregulated in GPAT2 overexpression lines (Fig. 3a). In the meantime, the transcript abundance of GPAT3 was not changed which localized into endoplasmic reticulum (Fig. 3b). Subsequently, the expression levels of few isoforms of LPAT and DGAT enzymes in TAG biosynthesis in order to examine the crucial role of GPAT2 on attracting the carbon metabolic precursors and convert them into the products [7, 16]. As shown in Fig. 3c–g, it was apparent that GPAT2 overexpression elicited the push-and-pull strategy by which the increased enzymatic activity of GPAT2 enhanced the substrate flux towards LPAT and DGAT. While at the same time, there was no difference noted for LPAT3 expression in transgenics, thereby identifying the crucial lipogenic enzymes. Reasoning the crucial lipogenic role of GPAT2 on increasing TAG content, we further sought to explore whether the DGAT2-associated TAG overproduction facilitated the expression of NADPH biosynthetic genes, as the lipid biosynthesis warrant adequate provision of NADPH as the reducing equivalents. Malic enzyme and glucose-6-phosphate dehydrogenase have been reported to play a critical role in providing lipogenic NADPH in oleaginous organisms [17, 18]. Thus, we determined the expression pattern of ME and G6PD in the overexpressing cells, which showed that expression of both the ME and G6PD was significantly increased in transgenic cells (Fig. 3h, i). Specifically, relative expression level of ME was found to be notably higher than G6PD in transgenics.

### Fatty acid acylation pattern revealed the unprecedented role of GPAT2 on governing fatty acid composition of TAGs

**Fig. 4:**
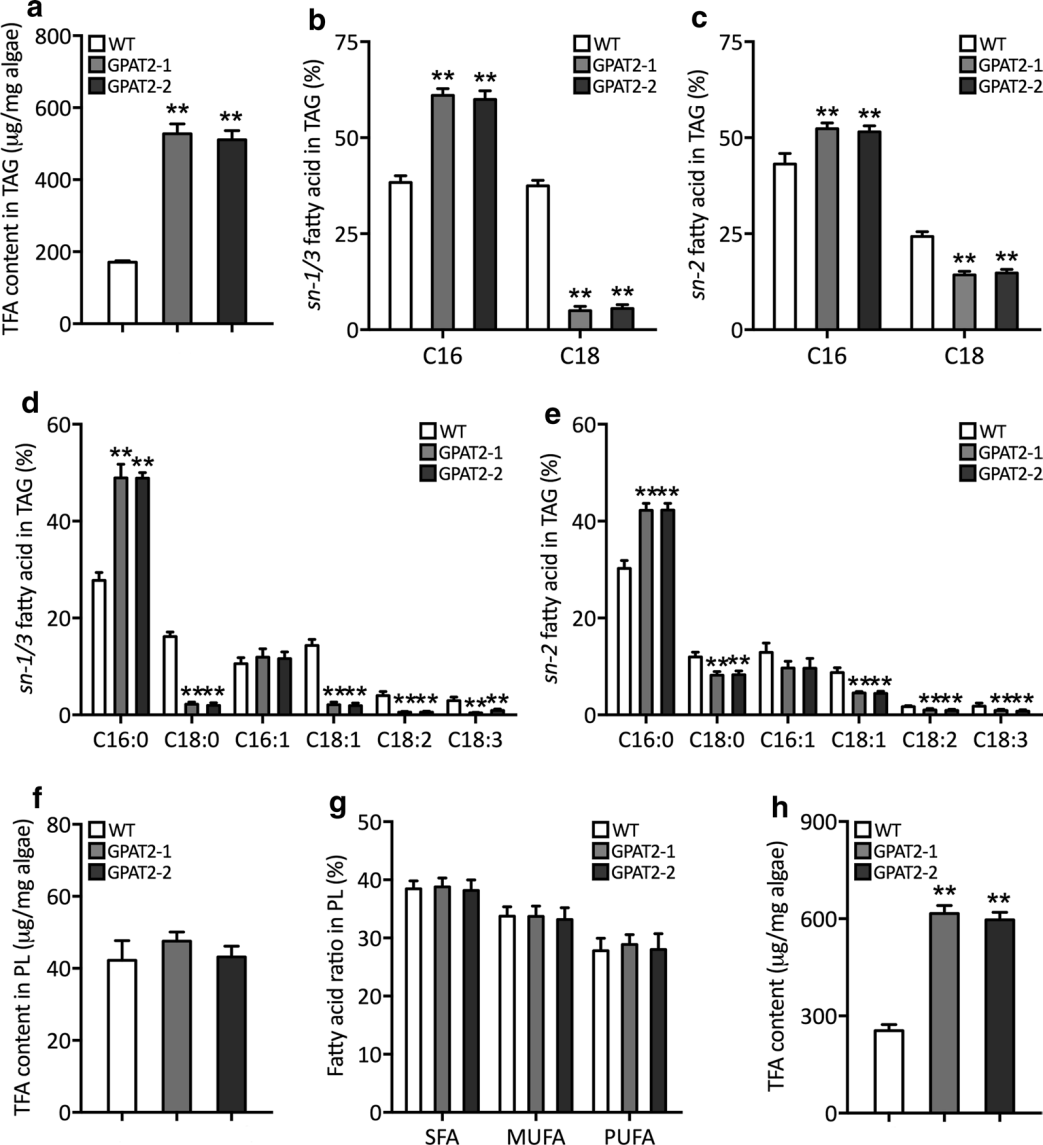
Analyses of fatty acid profile. **a** Total fatty acid content (µg/mg algae), **b** relative abundance of total C16 and C18 fatty acids accumulated in TAG at *sn*-1/3 position (%). **c** Relative abundance of total C16 and C18 fatty acids accumulated in TAG at *sn*-2 position (%). **d** Analysis of C16 and C18 fatty acid moieties in TAG at *sn*-1/3 positions. **e** Analysis of C16 and C18 fatty acid moieties in TAG at *sn*-2 positions. **f** Total fatty acid content in phospholipid (µg/mg algae). **g** Content of saturated, monounsaturated and polyunsaturated fatty acids in PL (%). **h** Total fatty acid content (µg/mg algae). Significant difference is indicated at the *p* < 0.05 (*) or *p* < 0.01 (**) level. Each value represents the mean ± SD (*n* = 3)

Given the increased lipid content, we further sought to elucidate the mechanism of GPAT2 on governing the fatty acid substrate specificity in pennate diatom *P. tricornutum*. As expected, GPAT2 overexpression significantly increased the total fatty acid content in TAGs of the transgenic cells (Fig. 4a). Interestingly, our fatty acid acylation pattern showed that C16 (*sn-*1/3 and *sn*-2) was found to be significantly higher in transgenic cells than WT, on the other hand, C18 (*sn-*1/3 and *sn*-2) was significantly lower in transgenic cells (Fig. 4b, c, Additional file 1: Tables S1–S2). Particularly, palmitic acid (C16:0) was found to be significantly increased in transgenic cells and palmitoleic acid (C16:1) was slightly increased in transgenic cells than that of WT (Fig. 4d, e). Stearic acid (C18:0), oleic acid (C18:1), linoleic acid (C18:2) and linolenic acid (C18:3) were found to be significantly decreased in overexpressing cells with relative to the WT cells (Fig. 4d, e). However, fatty acids in PL of transgenic lines were not changed compared to WT (Fig. 4f, g). Together, total fatty acid content was found to be significantly increased in transgenic cells by 2.3-fold than that of WT cells (Fig. 4h) and implied the crucial regulatory role of GPAT2 on governing TAG biosynthesis and fatty acid profile in *P. tricornutum*.

### GPAT2 overexpression confers hyposalinity and chilling tolerance in *P. tricornutum* besides its lipogenic role

GPAT has been well studied in oleaginous plants over the past few decades and these reports have uncovered the novel role of GAPT in conferring the tolerance to abiotic stress conditions such as salinity, lower temperature [11, 19]. However, data on their crucial mechanistic role other than lipogenesis are scant in microalgae. Given the altered fatty acid profile in the accumulated TAGs and the fact that altered saturation level on conferring stress tolerance to plants, we sought to uncover its role in abiotic stress associated mechanisms in transgenic *P. tricornutum*, particularly under hyposalinity (100, 90, 80 and 70%) and chilling stress conditions (25, 20, 15 and 10ºC). Our growth analyses of the overexpressing cells under hyposalinity and chilling stress showed that GPAT2 overexpressing cells exhibit better growth rate at 10ºC and 70% hyposalinity conditions (Additional file 1: Fig. S3). Specific growth rate was found to be significantly higher in the GPAT2 overexpressing cells under hyposalinity at 80% and 70%, though there was no significant difference between transgenic and WT at 100% and 90% hyposalinity conditions (Additional file 1: Fig. S3A). Congruently, gradual decrement in temperature resulted in a significant reduction of growth characteristics of WT, however, chilling stress conditions at 25 and 20ºC was not observed to show any difference in terms of growth rate between WT and transgenics (Additional file 1: Fig. S3C, D). Given the observed growth aberration in WT and the stress tolerance by the transgenics at 70% hyposaline and 10°C chilling conditions, we further evaluated the photosynthetic efficiency at this point of stress conditions. As shown in Additional file 1: Fig. S4, photosynthetic parameters were not impaired in the transgenics, whereas WT cells exhibited impaired photosynthetic rate.

**Fig. 5:**
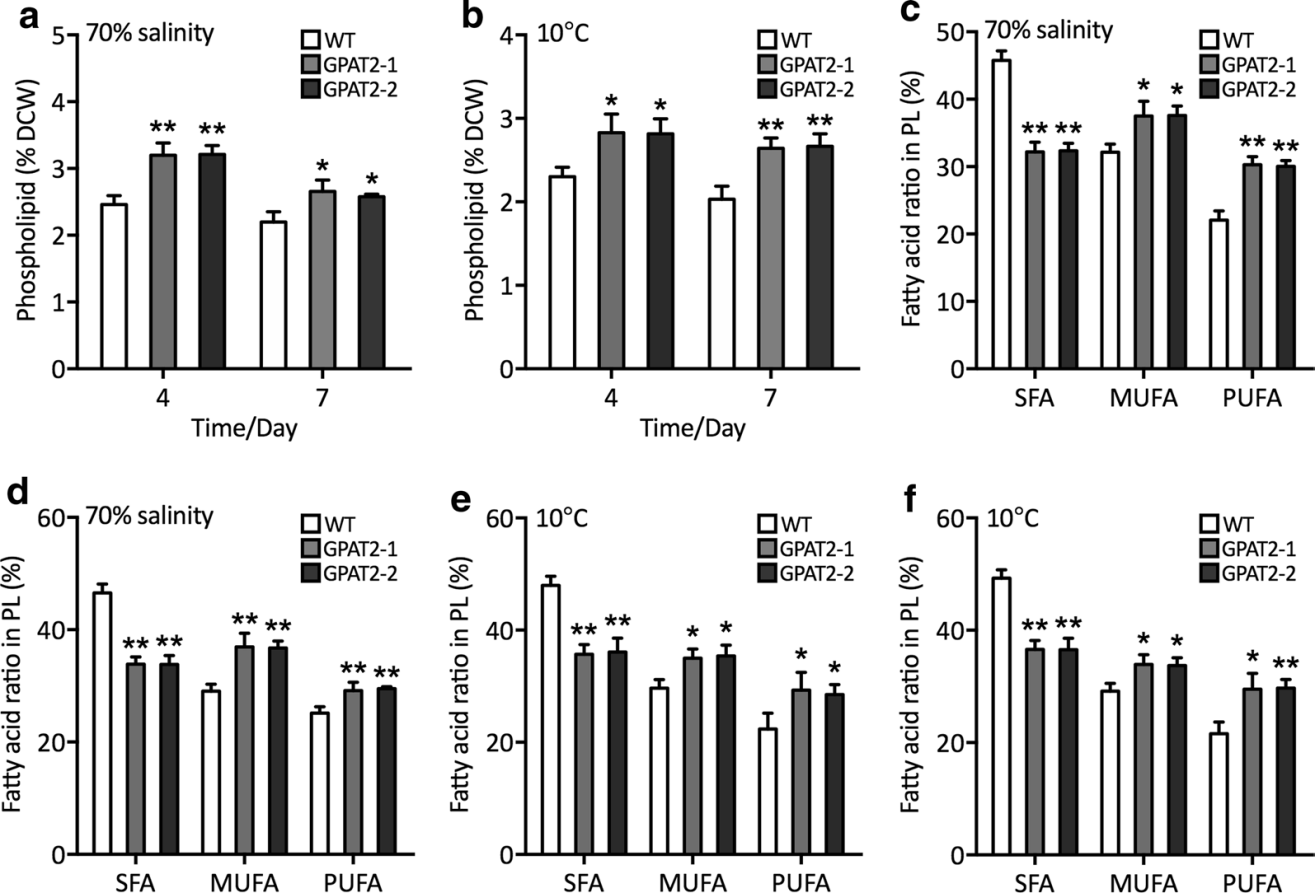
Analyses of phospholipids under hyposaline and chilling stress conditions. **a** Phospholipid content (% DCW) under 70% hyposaline conditions for 4- or 7-day treatment. **b** Phospholipid content (% DCW) at chilling condition (10 ºC) for 4- or 7-day treatment. Content of saturated, monounsaturated and polyunsaturated fatty acids in PL (% DCW) under 70% hyposaline conditions for **c** 4- or **d** 7-day treatment. Content of saturated, monounsaturated and polyunsaturated fatty acids in PL (% DCW) at chilling condition (10 ºC) for **e** 4- and **f** 7-day treatment. Significant difference is indicated at the *p* < 0.05 (*) or *p* < 0.01 (**) level. Each value represents the mean ± SD (*n* = 3)

**Fig. 6:**
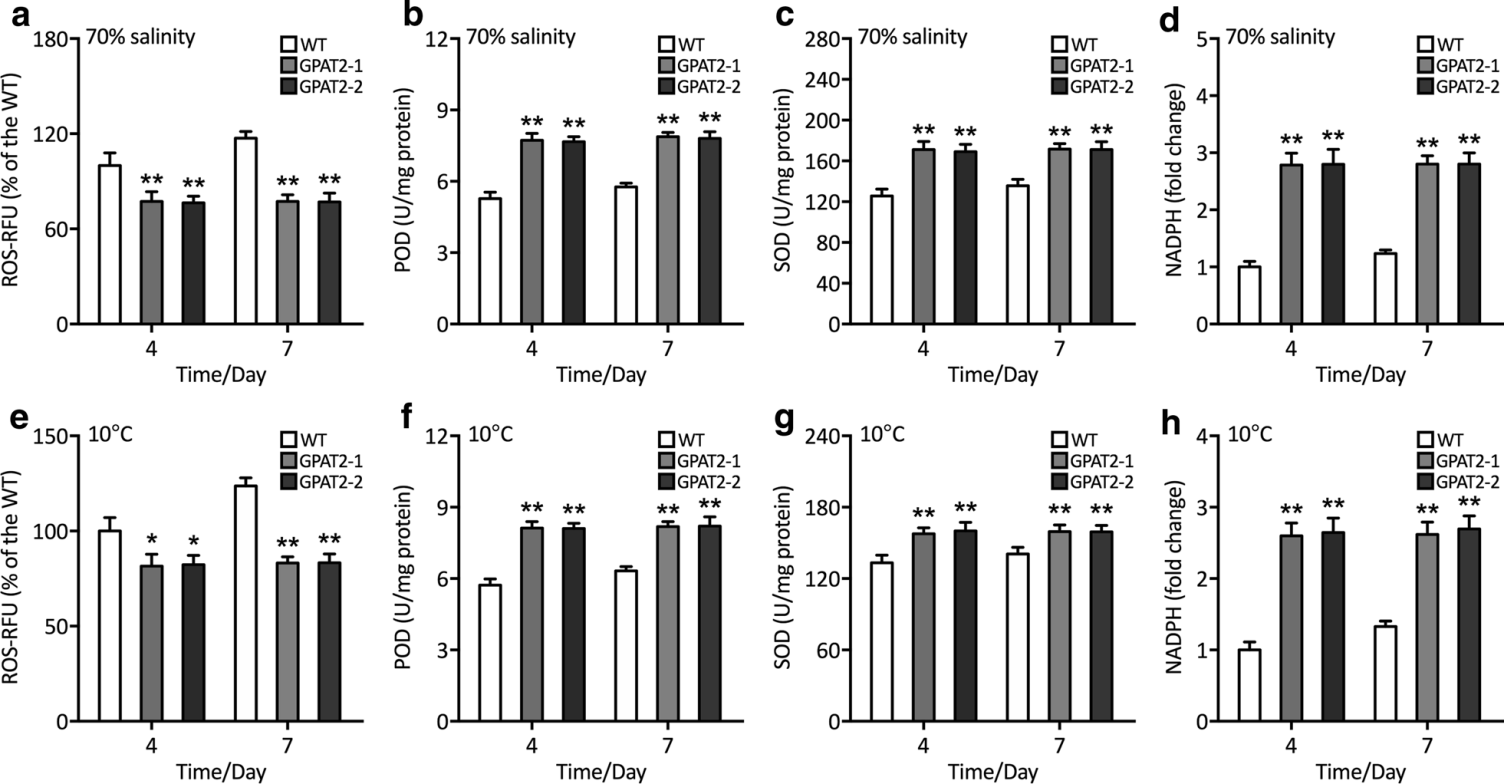
Analysis of antioxidant potential of algal cells. **a** Cellular ROS content as determined by H2DCFDA assay under 70% hyposaline condition for 4- or 7-day treatment. **b** Enzymatic assay of POD under 70% hyposaline condition for 4- or 7-day treatment. **c** Enzymatic assay of SOD under 70% hyposaline condition for 4- or 7-day treatment. **d** Cellular ROS content as determined by H2DCFDA assay under 70% hyposaline condition for 4- or 7-day treatment. **e** Cellular ROS content as determined by H2DCFDA assay at chilling condition (10 ºC) for 4- or 7-day treatment. **f** Enzymatic assay of POD at chilling condition (10 ºC) for 4- or 7-day treatment. **g** Enzymatic assay of SOD at chilling condition (10ºC) for 4- or 7-day treatment. **h** Cellular ROS content as determined by H2DCFDA assay at chilling condition (10 ºC) for 4- or 7-day treatment. Significant difference is indicated at the *p* < 0.05 (*) or *p* < 0.01 (**) level. Each value represents the mean ± SD (*n* = 3)

These results have provoked us to uncover the molecular mechanism underpinning this phenomenon. Lipid remodeling and alteration in fatty acid composition, particularly increased lipid unsaturation have been considered the potential acclimation strategy for the poikilothermic organisms to compensate the reduction in membrane fluidity due to the chilling stress [20]. We thus examined the fatty acid composition in the algal cells under hyposaline and chilling conditions. Intriguingly, phospholipid content was increased significantly in the transgenic cells subjected to both hyposaline and chilling stress conditions than that of the WT (Fig. 5a, b). In contrast, no such difference was observed in terms of lipid classes between WT and GPAT2 overexpressing cells under optimal conditions (Fig. 2h). It is interesting to note that during both hyposaline and chilling stress conditions unsaturated fatty acid content was found to be increased, whereas saturated fatty acid was decreased in transgenic cells than that of WT during 4th and 7th day of cultivation under stress treatments (Fig. 5c–f), thereby conferring tolerance to abiotic stress by facilitating the membrane fluidity [21]. These data implied the GPAT2 overexpression facilitated the lipid remodeling via the reduction of glycolipid, which could be used for the phospholipid generation, which is in accordance with the previous report [22]. While at the same time, glycolipids content was found to be decreased in the transgenic cells and no such reduction in glycolipid was observed in WT (Additional file 1: Fig. S5 and Fig. 2g). Besides, stress conditions could elicit the generation of reactive oxygen species, the key signaling molecules that regulate various metabolic pathways, however, lethal to macromolecules. Thus, determining the antioxidant potential of the cells is of importance to corroborate the stress associated role of the introduced transgene (Fig. 6). Antioxidant analyses showed that relative ROS content was decreased in transgenics than WT under both the stress conditions. Besides, the activities of the antioxidant enzymes such as peroxidase (POD), superoxide dismutase (SOD) were increased in transgenic cells than WT, implying that GPAT2 overexpression alleviated the stress imposed by the hyposaline and chilling stress [23]. Besides, NADPH content was also increased which corroborated the lipogenic role of GPAT2 in diatom. These data provided the crucial role of GPAT2 on conferring chilling and hyposaline stress tolerance conditions in *P. tricornutum* by unprecedentedly mediating crucial regulatory pathways.

## Discussion

Though oleaginous microalgae hold great potential as the candidate for biofuel production, large-scale production of biofuels in a commercially feasible manner is still impractical [24]. Among the most crucial techno-biological barriers that impede the algal commercial application, lack of industry-suitable algal strain with concurrent overproduction of lipids and biomass has been regarded as the major limiting factor. Metabolic engineering paves the way to obviate this bottleneck by precisely perturbing the metabolic target to enhance the desired product without affecting the cellular physiological parameters [25]. Nevertheless, lipid metabolism in microalgae is complex in nature due to the presence of regulatory factors at various levels, thereby enabling them recalcitrant to metabolic engineering [26]. Consequently, various previous studies have devoted tremendous research efforts to metabolically engineer oleaginous algal species, and studies showed that engineering the TAG pathway has resulted in promising results in microalgae [6, 7, 9, 16].

We thus attempted to overexpress the GPAT which catalyzes the first and committed step of acyl-CoA-dependent TAG biosynthesis in microalgae. In diatoms, five GPAT isoforms have been reported and the GPAT remains as an untapped metabolic target compared to the DGAT, the enzyme that catalyzes the final acylation step of DAG to TAG. In our previous study, we identified GPAT1 in *P. tricornutum*, which resulted in a significant elevation of neutral lipids by two-fold [6]. Similarly, dual overexpression of GPAT1 and LPAT1 resulted in a 2.3-fold increment in neutral lipids in *P. tricornutum* [9]. Previous report has shown that each isoform of GPAT exhibit crucial lipogenic role in mammals [27]. For instance, GPAT1 and GPAT4 are the major isoforms for glycerolipid biosynthesis in mammalian liver, and the lipidomic analyses of the mutant *Gpat4*^−/−^ and *Gpat1*^−/−^ mouse hepatocytes showed that GPAT1 metabolized de novo synthesized fatty acids into TAGs, and the results implied that GPAT1 and GPAT4 consume different pools of fatty acid for TAG biogenesis [28]. Adenoviral-mediated transformation of mitochondrial GPAT isoform in rat hepatocytes significantly increased the TAG biogenesis by reducing the fatty acid oxidation, while at the same time no compensatory effect on microsomal GPAT activity was observed [29]. Similarly, two isoforms of sunflower GPAT, namely *HaGPAT9-1* and *HaGPAT9-2* exhibited significantly different GPAT enzymatic activities despite their sequence similarities [30]. Collectively, these data have provoked the necessity to explore the mechanistic role of the GPAT isoforms in marine diatom in order to facilitate the filling-in gap process in the genetic improvement of microalgae.

In this study, we identified GPAT2 in the genome of *P. tricornutum* and characterized its role in lipid metabolic circuit. The identified GPAT2 exhibited close phylogeny relation with three isoforms of *B. napus*, whereas distantly related with other isoforms of *P. tricornutum* GPATs. GPAT4 isoforms from *B. napus* showed that BnGPAT4 played a crucial role in TAG biogenesis as well played a pivotal role in the development of embryos and reproductive organs which implied the possible role of GPAT2 on governing the developmental processes in *B. napus* besides its lipogenic role [31]. This data provided a clue to uncover the crucial unknown roles of GPAT2 by overexpressing it in the oleaginous marine diatom *P. tricornutum* besides the established lipogenesis. We further investigated the impact of GPAT2 overexpression on cellular growth and photosynthesis in *P. tricornutum*, which showed that GPAT2 overexpression did not impair cellular physiological properties. Our previous data have demonstrated that dual overexpression of GPAT1 and LPAT1 (AGPAT1) resulted in increased growth rate and photosynthetic efficiency [9]. On the other hand, overexpression of GPAT1 alone did not impede growth rate of the transgenic *P. tricornutum* [6]. Similarly, heterologous expression of GPAT from fresh water algae *Lobosphaera incisa* resulted in increased lipid content without impeding growth rate in transgenic *C. reinhardtii* [32]. Overexpression of both GPAT1 and GPAT2 in red algae *Cyanidioschyzon merolae* increased lipid content with negatively affecting the algal growth rate [14]. Collectively, overexpression of GPAT2 did not impede the growth and photosynthetic rate in *P. tricornutum*, which is in line with previous reports.

Thereafter, impact of GPAT2 overexpression on primary metabolite content was determined which showed that carbohydrate and protein content were decreased, whereas lipid content was found to be increased. Similarly, overexpression of LPAT1 altered the primary metabolite content with reduced carbohydrate and protein content and increased lipid content in transgenic *P. tricornutum* [7]. Moreover, dual expression of GPAT1 and LPAT1 resulted in similar consequences in terms of primary metabolite content alteration transgenic cells [9]. These results implied that GPAT1 overexpression facilitated the redirection of carbon metabolic precursors, energy and reducing equivalents towards lipid biosynthesis from carbohydrate metabolism, which is consistent with the previous reports. To investigate the regulatory role of GPAT1 on orchestrating the other key enzymes in TAG biosynthesis, we determined the relative transcript level of LPAT and DGAT in transgenic cells. Results revealed that GPAT2 overexpression increased the expression levels of other key genes such as LPAT and DGAT in the TAG pathway. This consequence might be due to the increased abundance of product (phosphatidic acid and diacylglycerol) in response to the increased GPAT2 activity, which in turn act as the substrate for subsequent enzymes that resulted in the increased expression of LPAT and DGAT in transgenic microalgae [7]. Previous studies have shown that plant GPATs might not execute strong substrate preference unlike LPAT [33], however, recent reports have suggested that GPAT could result in enrichment of specific fatty acid moieties in TAGs [34, 35]. Heterologous expression of *L. incisa* resulted in enhanced TAG production with the altered fatty acid profile, particularly sharp increment in C16:0 fatty acid moieties in TAG [32]. Our results showed that GPAT2 overexpression significantly altered the fatty acid profile and C16:0 was found to be the predominant fatty acid moiety in the accumulated TAGs in transgenic cells which is consistent with the previous reports.

**Fig. 7:**
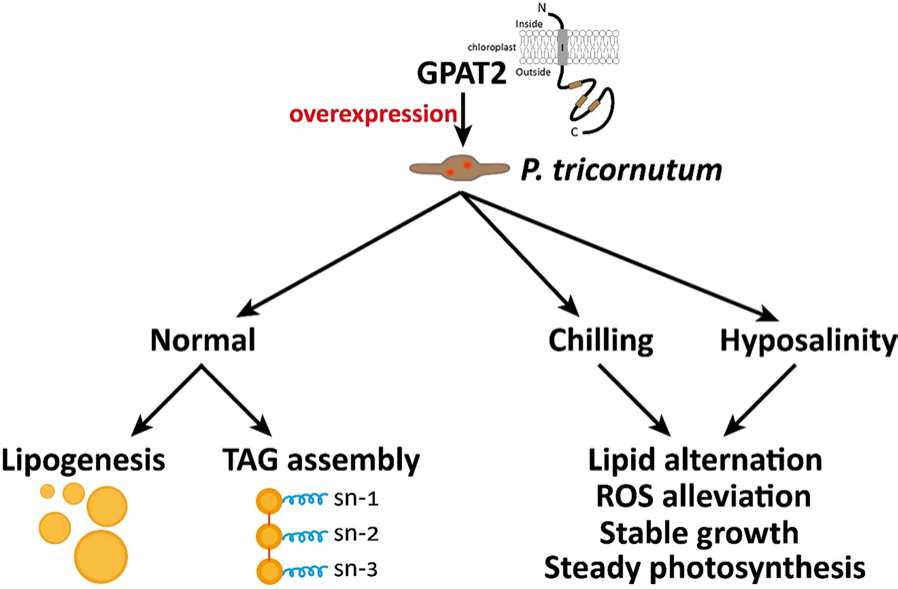
Schematic illustration of the mechanistic role of GPAT2 in *P. tricornutum.* Overexpression of GPAT2 enhanced lipogenesis with engineered fatty acid profile under optimum cultivation conditions, on the other hand, GPAT2 overexpression confers stress tolerance to chilling and hyposaline stress conditions

Previous reports have demonstrated that GPATs from *B. napus* to which our GPAT2 from *P. tricornutum* showed close phylogenetic relations have been demonstrated to carry out crucial physiological role in *B. napus*. Similarly, GPAT of *Arabidopsis*, a closely related species of *B. napus* involved in various physiological mechanisms in addition to their known lipid biosynthesis [36, 37]. These data provided a valuable insight into the unknown functional mechanisms of GPATs underlying the cellular physiological processes besides the known TAG biosynthesis. Previous studies have reported the efficacy of GPATs on conferring abiotic stress tolerance to higher plants, nevertheless no such data is available in oleaginous diatom. Given the increased lipid content and altered fatty acid profile in GPAT2 overexpressing cells, we speculated that GPAT2 overexpression might confer some additional physiological functions to the diatom. Thus, we subjected the transgenic cells to hyposaline and chilling stress conditions to uncover the possible role of GPAT2 on conferring tolerance to these stress conditions. GPAT2 overexpressing cells showed resistance to these stress conditions than that of the WT cells. Particularly, GPAT2 overexpression increased the growth and photosynthetic activity of the transgenic cells under 70% hyposaline and 10ºC chilling stress conditions. Overexpression of tomato GPAT resulted in tolerance to chilling stress conditions which was implied by the reduced relative electrolyte leakage and improved photosynthetic parameters, probably by altering the fatty acid profile of the TAGs and consequently alter the membrane fluidity in transgenic tomatoes [38]. Heterologous expression of GPAT from sweet pepper resulted in enhanced photosynthetic activity of transgenic tobacco plants under high temperature by enhancing the saturation extent of thylakoid membrane lipids in transgenic tobacco plants [19]. Similarly, heterologous expression of GPAT from *Suaeda salsa* resulted in enhanced salt tolerance in transgenic *Arabidopsis* by upholding the unsaturated fatty acid content, thereby protecting the photosynthetic machinery in transgenic plants [11]. Our results showed that unprecedented role of GPAT2 in increasing TAG content, alteration of fatty acid profile and upholding of photosynthetic machinery enhanced the TAG content significantly and conferred resistance to abiotic stress conditions in marine diatom (Fig. 7).

## Conclusion

TAG pathway rewiring has emerged as the promising strategy to improve algal commercial potential, however, existing intricacies in lipogenesis constrain this approach. We perturbed the TAG pathway by targeting GPAT2, the first committed enzyme of TAG pathway. GPAT2 overexpression significantly increased TAG with engineered fatty acid profile by 2.9-fold than WT, thereby conferring abiotic stress tolerance to engineered cells. Furthermore, GPAT2 overexpression elicited the push-and-pull strategy by increasing the abundance of substrates for the subsequent metabolic enzymes. Together, this report exemplifies a feasible strategy to empower algal commercial potential through unprecedented roles of the GPAT2.

## Methods

### Strain and culture conditions

*Phaeodactylum tricornutum* CCMP-2561 was purchased from the Provasoli-Guillard National Center for Marine Algae and Microbiota (East Boothbay, USA) and cultivated into filtered natural seawater added with f/2 medium without Si in artificial climate incubator with 200 μmol photons m^−2^ s^−1^ cold light irradiance at 20 ± 0.5 °C under a 12 h: 12 h light/dark cycle. In addition, cells or engineered cells grown under normal or stress conditions were harvested at 4400 rpm for 10 min for further experiments.

### Plasmid construction and transformation

The plasmid was constructed according to the protocol mentioned previously [39]. Briefly, plasmid pHY18 containing the chloramphenicol acetyltransferase antibiotic gene (CAT) controlled by the nitrate reductase (NR) promoter and terminator from *P. tricornutum* was used as the vector. Target gene GPAT2 was inserted into pHY18 controlled by the fucoxanthin chlorophyll a/c-binding protein C (fcpC) promoter and fcpA terminator to form recombinant plasmid pHY18-GPAT2. The purified recombinant plasmid amplified from *Escherichia coli* were linearized and then transformed into diatom cells by electroporation following the previous protocol [9]. After electroporation, cells were recovered in fresh liquid f/2 medium without antibiotics for at least 48 h and then transferred into solid f/2 medium with chloramphenicol (250 mg L^−1^) for 4 weeks. Total RNA from engineered and WT strains was isolated by Plant RNA kit (Omega, USA) and then transcribed into cDNA by HiScript II Q RT SuperMix for qPCR (Vazyme, China). qRT-PCR was accomplished by eight-strip tube with 20 μL volume system and performed in CFX Connect™ Real-Time PCR Detection System (Bio-rad, USA). The relative transcriptional expression level of microalgae was calculated by the 2^−ΔΔCt^ method normalized to internal gene *β-actin*. The enzymatic activity of GPAT2 was determined by Plant GPAT Activity Spectrophotometry Assay kit (Bangyi, China) according to the manufacturer’s instruction.

### Photosynthesis and growth determination

Chlorophyll *α* content was measured by Agilent 1200 HPLC system with a Symmetry C8 column as described before [9]. Photochemical efficiency of photosystem II (Fv/Fm) and electron transport rate (ETR) were determined by PhytoPAM Phytoplankton Analyzer (Walz, Germany). Non-photochemical quenching (NPQ) was calculated by the following formula: NPQ = (Fm – Fm’)/Fm’. Cell growth rate was measured by growth curve method which is determined by direct counting of the algal cells every day using Neubauer chamber under light microscope as described elsewhere [9]. Specific growth rate of both the transgenic and WT algal cells was determined as described elsewhere [40].

### Primary metabolite analysis

Total protein was isolated from microalgae by RIPA lysis buffer (Beyotime, China) added with phenylmethylsulfonyl fluoride (PMSF, Beyotime, China) and quantified by BCA protein quantification kit (Beyotime, China) according to the standard curve of bovine serum albumin. Total carbohydrate content from the algal cells was estimated by phenol–sulfuric acid method and calculated according to the standard curve of glucose [9].

Relative neutral lipid (NL) content was determined qualitatively by Nile-red fluorometric assay method [7]. Briefly, 3 mL cells were added with Nile-red solution (0.1 mg/mL dissolved in acetone) and lucifugally incubated for 20 min at 37 °C. Thereafter stained cells were transferred into 96-well plate for fluorescence intensity detection by microplate reader (Bio-Tek, USA) at a 480 nm excitation wavelength and a 592 nm emission wavelength. Besides, total lipid content was extracted following the modified protocol of Bligh and Dyer [41] and the lipid content of cell dry weight was determined gravimetrically [7]. Moreover, total lipid was fractionated into NL, phospholipid (PL) and glycolipid (GL) by solid-phase extraction (SPE) column with pre-packed silica cartridges (500 mg, 6 cc Sep-Pak, Waters, USA). After pre-flushing with 4 mL chloroform to the column, total lipid was sequentially fractionated by solvents of chloroform, acetone and methanol to collect NL, GL and PL, respectively. Each fractionated lipid was dried by N_2_ stream flow and gravimetrically determined [7].

TAG was separated from total lipid by thin-layer chromatography on a silica plate (Merck, Germany) with the developing mixture solvent (hexane/diethyl ether/acetic acid, 85:15:1, *v*/*v*/*v*) [7]. The isolated TAG was visualized in silica plate using iodine vapor and the bands were scraped from the TAG-localized silica plate into 1.5-mL Eppendorf tube with chloroform. The solution was vortexed and then centrifuged at 12,000 × *g* for 10 min. Finally, the supernatant was dried under a nitrogen steam and TAG content was determined gravimetrically following the protocols provided elsewhere [7].

### Determination of expression levels of TAG and NADPH synthetic genes

The relative mRNA level of several key lipogenic genes (*GPAT1*, *GPAT3*, *LPAT1*, *LPAT2*, *LPAT3*, *DGAT2A*, *DGAT2D*, *G6PD*, *ME*) was determined by quantitative real-time PCR (qPCR) using AceQ qPCR SYBR Green Master Mix (Vazyme, China) and performed on CFX96 real-time PCR detection system [7]. The relative mRNA level of these genes was determined by the 2^−ΔΔCt^ method after normalization to the endogenous control gene *β*-*actin*. Three biological replicates were performed. All primers for qPCR assays are listed in Additional file 1: Table S3.

### Analysis of fatty acid composition

Fatty acids in total lipid, TAG and PL were transesterified and the fatty acid composition was analyzed as FAMEs using gas chromatography–mass spectrometry (GC–MS) [7, 9]. The content of fatty acid was analyzed by gas chromatography–mass spectrophotometer (GC–MS) equipped with the spectrum library. The peak area of fatty acid esters was normalized by the reference standard methyl nonadecylate (Aladdin, China). For positional analysis of TAG, TAG was firstly dissolved into PBS containing Triton X-100 and emulsified for 6 h by *R. arrhizus* lipase (Sigma-Aldrich, USA). The emulsified reaction was blocked by 1 mL chloroform–methanol (1:1, *v*/*v*) mixture. Fatty acids were separated by TLC and scraped the isolated free fatty acids and monoacylglycerol for transesterification described above.

### Chilling and hyposaline treatment

For chilling and hyposaline treatment, cells were firstly treated under nitrogen depleted (–N) condition for 48 h to synchronize physiological statues. The collected microalgae by centrifugation were treated with different saline concentration (70%-100%, 100% referred to the original salinity) and temperature (10–25 °C) for 7 days. Meanwhile, cell density was counted described above and specific growth rate was calculated by the equation described above. Additionally, selected hyposalinity and temperature were used for next experiments. The chlorophyll *α*, NPQ, ETR and Fv/Fm of cells under selected hyposalinity and temperature were measured described above. PL was isolated firstly and then transesterified according to the protocol described above. Fatty acid content and profile of PL was analyzed by GC–MS.

Reactive oxygen species (ROS) of *P. tricornutum* during the selected hyposalinity and temperature was measured using cell-permeable fluorogenic probe 2′,7′-dichlorodihydrofluorescein diacetate (DCFH-DA, Beyotime, China). Briefly, cells were stained with 1 mM DCFH-DA at a ratio of 1:100 (*v*/*v*) and then incubated at 37 °C for 30 min in darkness. Cells were washed by PBS for thrice and detected by microplate reader at a 488 nm excitation wavelength and a 500–600 nm emission wavelength [42]. The oxidative enzymes such as peroxidase (POD) and superoxide dismutase (SOD) were determined by commercial kits retrieved from Beyotime. In addition, NADPH content of microalgae was determined by Amplite™ colorimetric NADPH assay kit according to the manufacturer’s instructions (AAT Bioquest, USA) [43].

### Statistical analysis

All experiments in this study were performed at least in triplicate, while the data were shown as mean ± SD. The statistical tests were carried out by SPSS statistical package 19.0. Student’s *t*-test were performed to compared the engineered and WT strains. All results were considered to be significantly different at *p* < 0.05 (*) or *p* < 0.01 (**).

## Supplementary information

**Additional file 1: Fig. S1.** In silico analyses of GPAT2 sequence. **(A)** Protein structure of GPAT2. **(B)** Phylogeny analysis of GPAT2 from the deduced amino acid sequence of GPAT2 and GPAT of various organisms. Phylogenetic tree was constructed by MEGA using Neighbor joining method. Percentage of the replicate trees where the taxa clustered together in the bootstrap value (500 replicates) are provided in the branches. Arrow mark denotes the GPAT2 of *P. tricornutum*. **Fig. S2.** Analyses of photosynthetic parameters in transgenics. **(A)** Maximum quantum yield of photosystem II as indicated by Fv/Fm. **(B)** Determination of chlorophyll α content (pg/cell). **(C)** Non-photo chemical quenching (NPQ). **(D)** Electron transport rate (ETR) (µmol e^-^m^-2^s^-1^). Significant difference between WT and transgenics is indicated at the *p* < 0.05 (*) or *p* < 0.01 (**) level. Each value represents the mean ± SD (*n* = 3). **Fig. S3.** Characterization of physiological parameters under hyposaline and chilling conditions. Specific growth rate **(A)** and growth curve **(B)** under hyposaline conditions. Specific growth rate **(C)** and growth curve **(D)** at chilling condition (10 ºC). Significant difference between WT and transgenics is indicated at the *p* < 0.05 (*) or *p* < 0.01 (**) level. Each value represents the mean ± SD (*n* = 3). **Fig. S4.** Characterization of photosynthetic parameters under 70 % hyposaline and 10 ºC. Determination of **(A)** Chlorophyll α content, **(B)** NPQ, **(C)** ETR (µmol e^-^m^-2^s^-1^) and **(D)** Fv/Fm under 70% hyposaline conditions. Determination of **(E)** Chlorophyll α content, **(F)** NPQ, **(G)** ETR (µmol e^-^m^-2^s^-1^) and **(H) **Fv/Fm at 10 ºC. Significant difference between WT and transgenics is indicated at the *p* < 0.05 (*) or *p* < 0.01 (**) level. Each value represents the mean ± SD (*n* = 3). **Fig. S5.** Analysis of glycolipids in transgenic cells under hyposaline and chilling stress conditions.** (A)** Glycolipid content (% DCW) under 70% hyposaline conditions. **(B)** Glycolipid content (% DCW) under chilling condition (10 ºC). Significant difference between WT and transgenics is indicated at the *p* < 0.05 (*) or *p* < 0.01 (**) level. Each value represents the mean ± SD (*n* = 3). **Table S1.** Proportion of C16 and C18 fatty acids in TAG at *sn*-1/3 position. **Table S2.** Proportion of C16 and C18 fatty acids in TAG at *sn*-2 position. **Table S3.** Primers used in this study.

## Data Availability

All data supporting the conclusions of this article are included within the article and in Additional files.

## Abbreviations

TAG: Triacylglycerides
GPAT: Glycerol-3-phosphate acyltransferase
DGAT: Diacylglycerol acyltransferase
AGPAT: 1-Acyl-sn-glycerol-3-phosphate acyltransferase
ME: Malic enzyme
WT: Wild type
G6PD: Glucose-6-phosphate dehydrogenase
POD: Peroxidase
SOD: Superoxide dismutase
ROS: Reactive oxygen species
NR: Nitrate reductase
fcp: Fucoxanthin chlorophyll a/c-binding protein
NPQ: Non-photochemical quenching

## References

[1] Haslam RP, Hamilton ML, Economou CK, Smith R, Hassall KL, Napier JA (2020). Overexpression of an endogenous type 2 diacylglycerol acyltransferase in the marine diatom *Phaeodactylum tricornutum* enhances lipid production and omega-3 long-chain polyunsaturated fatty acid content. Biotechnol Biofuels.

[2] Kim M, Park BG, Kim E-J, Kim J, Kim B-G (2019). In silico identification of metabolic engineering strategies for improved lipid production in *Yarrowia lipolytica* by genome-scale metabolic modeling. Biotechnol Biofuels.

[3] Büchel C (2019). How diatoms harvest light. Science.

[4] Butler T, Kapoore RV, Vaidyanathan S (2020). *Phaeodactylum tricornutum*: a diatom cell factory. Trends Biotechnol.

[5] Yang Z-K, Niu Y-F, Ma Y-H, Xue J, Zhang M-H, Yang W-D (2013). Molecular and cellular mechanisms of neutral lipid accumulation in diatom following nitrogen deprivation. Biotechnol Biofuels.

[6] Niu Y-F, Wang X, Hu D-X, Balamurugan S, Li D-W, Yang W-D (2016). Molecular characterization of a glycerol-3-phosphate acyltransferase reveals key features essential for triacylglycerol production in *Phaeodactylum tricornutum*. Biotechnol Biofuels.

[7] Balamurugan S, Wang X, Wang H-L, An C-J, Li H, Li D-W (2017). Occurrence of plastidial triacylglycerol synthesis and the potential regulatory role of AGPAT in the model diatom *Phaeodactylum tricornutum*. Biotechnol Biofuels.

[8] Yamaoka Y, Achard D, Jang S, Legéret B, Kamisuki S, Ko D (2016). Identification of a *Chlamydomonas* plastidial 2-lysophosphatidic acid acyltransferase and its use to engineer microalgae with increased oil content. Plant Biotechnol J.

[9] Wang X, Dong H-P, Wei W, Balamurugan S, Yang W-D, Liu J-S (2018). Dual expression of plastidial GPAT1 and LPAT1 regulates triacylglycerol production and the fatty acid profile in *Phaeodactylum tricornutum*. Biotechnol Biofuels.

[10] Li X, Liu P, Yang P, Fan C, Sun X (2018). Characterization of the glycerol-3-phosphate acyltransferase gene and its real-time expression under cold stress in *Paeonia lactiflora* Pall. PLoS ONE.

[11] Sui N, Tian S, Wang W, Wang M, Fan H (2017). Overexpression of glycerol-3-phosphate acyltransferase from *Suaeda salsa* improves salt tolerance in *Arabidopsis*. Front Plant Sci.

[12] Li Y, Han D, Hu G, Sommerfeld M, Hu Q (2010). Inhibition of starch synthesis results in overproduction of lipids in *Chlamydomonas reinhardtii*. Biotechnol Bioeng.

[13] Radakovits R, Eduafo PM, Posewitz MC (2011). Genetic engineering of fatty acid chain length in *Phaeodactylum tricornutum*. Metab Eng.

[14] Fukuda S, Hirasawa E, Takemura T, Takahashi S, Chokshi K, Pancha I (2018). Accelerated triacylglycerol production without growth inhibition by overexpression of a glycerol-3-phosphate acyltransferase in the unicellular red alga *Cyanidioschyzon merolae*. Sci Rep.

[15] Muñoz CF, Weusthuis RA, D’Adamo S, Wijffels RH (2019). Effect of single and combined expression of lysophosphatidic acid acyltransferase, glycerol-3-phosphate acyltransferase, and diacylglycerol acyltransferase on lipid accumulation and composition in *Neochloris oleoabundans*. Front Plant Sci.

[16] Wei H, Shi Y, Ma X, Pan Y, Hu H, Li Y (2017). A type-I diacylglycerol acyltransferase modulates triacylglycerol biosynthesis and fatty acid composition in the oleaginous microalga *Nannochloropsis oceanica*. Biotechnol Biofuels.

[17] Osada K, Maeda Y, Yoshino T, Nojima D, Bowler C, Tanaka T (2017). Enhanced NADPH production in the pentose phosphate pathway accelerates lipid accumulation in the oleaginous diatom *Fistulifera solaris*. Algal Res.

[18] Ratledge C (2014). The role of malic enzyme as the provider of NADPH in oleaginous microorganisms: a reappraisal and unsolved problems. Biotechnol Lett.

[19] Yan K, Chen N, Qu Y-Y, Dong X-C, Meng Q-W, Zhao S-J (2008). Overexpression of sweet pepper glycerol-3-phosphate acyltransferase gene enhanced thermotolerance of photosynthetic apparatus in transgenic tobacco. J Integr Plant Biol.

[20] Mironov KS, Sidorov RA, Trofimova MS, Bedbenov VS, Tsydendambaev VD, Allakhverdiev SI (2012). Light-dependent cold-induced fatty acid unsaturation, changes in membrane fluidity, and alterations in gene expression in *Synechocystis*. Biochim Biophys Acta Bioenergetics.

[21] Scotti-Campos P, Pais IP, Partelli FL, Batista-Santos P, Ramalho JC (2014). Phospholipids profile in chloroplasts of *Coffea* spp. genotypes differing in cold acclimation ability. J Plant Physiol..

[22] Gao X, Liu W, Mei J, Xie J (2019). Quantitative analysis of cold stress inducing lipidomic changes in *Shewanella putrefaciens* using UHPLC-ESI-MS/MS. Molecules.

[23] Yan L, Shah T, Cheng Y, LÜ Y, Zhang XK, Zou XL (2019). Physiological and molecular responses to cold stress in rapeseed (*Brassica napus* L.). J Integr Agric..

[24] Zhang T-Y, Hu H-Y, Wu Y-H, Zhuang L-L, Xu X-Q, Wang X-X (2016). Promising solutions to solve the bottlenecks in the large-scale cultivation of microalgae for biomass/bioenergy production. Renew Sustain Energy Rev.

[25] Jagadevan S, Banerjee A, Banerjee C, Guria C, Tiwari R, Baweja M (2018). Recent developments in synthetic biology and metabolic engineering in microalgae towards biofuel production. Biotechnol Biofuels.

[26] Liu B, Benning C (2013). Lipid metabolism in microalgae distinguishes itself. Curr Opin Biotechnol.

[27] Lu B, Jiang YJ, Kim P, Moser A, Elias PM, Grunfeld C (2010). Expression and regulation of GPAT isoforms in cultured human keratinocytes and rodent epidermis. J Lipid Res.

[28] Wendel AA, Cooper DE, Ilkayeva OR, Muoio DM, Coleman RA (2013). Glycerol-3-phosphate acyltransferase (GPAT)-1, but not GPAT4, incorporates newly synthesized fatty acids into triacylglycerol and diminishes fatty acid oxidation. J Biol Chem.

[29] Lindén D, William-Olsson L, Rhedin M, Asztély A-K, Clapham JC, Schreyer S (2004). Overexpression of mitochondrial GPAT in rat hepatocytes leads to decreased fatty acid oxidation and increased glycerolipid biosynthesis. J Lipid Res.

[30] Payá-Milans M, Aznar-Moreno JA, Balbuena TS, Haslam RP, Gidda SK, Pérez-Hormaeche J (2016). Sunflower HaGPAT9-1 is the predominant GPAT during seed development. Plant Sci.

[31] Chen X, Chen G, Truksa M, Snyder CL, Shah S, Weselake RJ (2014). Glycerol-3-phosphate acyltransferase 4 is essential for the normal development of reproductive organs and the embryo in *Brassica napus*. J Exp Bot.

[32] Iskandarov U, Sitnik S, Shtaida N, Didi-Cohen S, Leu S, Khozin-Goldberg I (2016). Cloning and characterization of a GPAT-like gene from the microalga *Lobosphaera incisa* (Trebouxiophyceae): overexpression in *Chlamydomonas reinhardtii* enhances TAG production. J Appl Phycol.

[33] Suh M, Schultz DJ, Ohlrogge JB (2002). What limits production of unusual monoenoic fatty acids in transgenic plants?. Planta.

[34] Cattaneo ER, Pellon-Maison M, Rabassa ME, Lacunza E, Coleman RA, Gonzalez-Baro MR (2012). Glycerol-3-phosphate acyltransferase-2 is expressed in spermatic germ cells and incorporates arachidonic acid into triacylglycerols. PLoS ONE.

[35] Iskandarov U, Khozin-Goldberg I, Cohen Z (2011). Selection of a DGLA-producing mutant of the microalga *Parietochloris incisa*: identification of mutation site and expression of VLC-PUFA biosynthesis genes. Appl Microbiol Biotechnol.

[36] Li X-C, Zhu J, Yang J, Zhang G-R, Xing W-F, Zhang S (2012). Glycerol-3-phosphate acyltransferase 6 (GPAT6) is important for tapetum development in *Arabidopsis* and plays multiple roles in plant fertility. Mol Plant.

[37] Xue M, Guo T, Ren M, Wang Z, Tang K, Zhang W (2019). Constitutive expression of chloroplast glycerol-3-phosphate acyltransferase from *Ammopiptanthus mongolicus* enhances unsaturation of chloroplast lipids and tolerance to chilling, freezing and oxidative stress in transgenic *Arabidopsis*. Plant Physiol Biochem.

[38] Sui N, Li M, Zhao S-J, Li F, Liang H, Meng Q-W (2007). Overexpression of glycerol-3-phosphate acyltransferase gene improves chilling tolerance in tomato. Planta.

[39] Xue J, Niu Y-F, Huang T, Yang W-D, Liu J-S, Li H-Y (2015). Genetic improvement of the microalga *Phaeodactylum tricornutum* for boosting neutral lipid accumulation. Met Eng.

[40] Sandnes JM, Källqvist T, Wenner D, Gislerød HR (2005). Combined influence of light and temperature on growth rates of *Nannochloropsis oceanica*: linking cellular responses to large-scale biomass production. J Appl Phycol.

[41] Bligh EG, Dyer WJ (1959). A rapid method of total lipid extraction and purification. Can J Biochem Physiol.

[42] Wang X, Luo S-W, Luo W, Yang W-D, Liu J-S, Li H-Y (2019). Adaptive evolution of microalgal strains empowered by fulvic acid for enhanced polyunsaturated fatty acid production. Bioresour Technol.

[43] Wang X, Balamurugan S, Liu S-F, Zhang M-M, Yang W-D, Liu J-S (2020). Enhanced polyunsaturated fatty acid production using food wastes and biofuels byproducts by an evolved strain of *Phaeodactylum tricornutum*. Bioresour Technol.

