# Supplementary material for: TAG pathway engineering via GPAT2 concurrently potentiates abiotic stress tolerance and oleaginicity in Phaeodactylum tricornutum
Source: Biotechnol Biofuels. 2020 Sep 14;13:160. doi: 10.1186/s13068-020-01799-5 (PMC7491103; doi:10.1186/s13068-020-01799-5)
Supplement: Supplementary file 1 — Additional file 1: Fig. S1. In silico analyses of GPAT2 sequence. (A) Protein structure of GPAT2. (B) Phylogeny analysis of GPAT2 from the deduced amino acid sequence of GPAT2 and GPAT of various organisms. Phylogenetic tree was constructed by MEGA using Neighbor joining method. Percentage of the replicate trees where the taxa clustered together in the bootstrap value (500 replicates) are provided in the branches. Arrow mark denotes the GPAT2 of P. tricornutum. Fig. S2. Analyses of photosynthetic parameters in transgenics. (A) Maximum quantum yield of photosystem II as indicated by Fv/Fm. (B) Determination of chlorophyll α content (pg/cell). (C) Non-photo chemical quenching (NPQ). (D) Electron transport rate (ETR) (µmol e-m-2s-1). Significant difference between WT and transgenics is indicated at the p < 0.05 (*) or p < 0.01 (**) level. Each value represents the mean ± SD (n = 3). Fig. S3. Characterization of physiological parameters under hyposaline and chilling conditions. Specific growth rate (A) and growth curve (B) under hyposaline conditions. Specific growth rate (C) and growth curve (D) at chilling condition (10 ºC). Significant difference between WT and transgenics is indicated at the p < 0.05 (*) or p < 0.01 (**) level. Each value represents the mean ± SD (n = 3). Fig. S4. Characterization of photosynthetic parameters under 70 % hyposaline and 10 ºC. Determination of (A) Chlorophyll α content, (B) NPQ, (C) ETR (µmol e-m-2s-1) and (D) Fv/Fm under 70% hyposaline conditions. Determination of (E) Chlorophyll α content, (F) NPQ, (G) ETR (µmol e-m-2s-1) and (H) Fv/Fm at 10 ºC. Significant difference between WT and transgenics is indicated at the p < 0.05 (*) or p < 0.01 (**) level. Each value represents the mean ± SD (n = 3). Fig. S5. Analysis of glycolipids in transgenic cells under hyposaline and chilling stress conditions. (A) Glycolipid content (% DCW) under 70% hyposaline conditions. (B) Glycolipid content (% DCW) under chilling condition (10 ºC). Significant [file 13068_2020_1799_MOESM1_ESM.docx]

**Additional material**

**TAG pathway engineering via GPAT2 concurrently potentiates abiotic stress tolerance and oleaginicity in *Phaeodactylum tricornutum***

Xiang Wang ^a,b,1^, Si-Fen Liu ^a,1^, Ruo-Yu Li ^a^, Wei-Dong Yang ^a^, Jie-Sheng Liu ^a^, Carol Sze Ki Lin ^b^, Srinivasan Balamurugan ^a,c,*^, Hong-Ye Li ^a,*^

^a^ Key Laboratory of Eutrophication and Red Tide Prevention of Guangdong Higher Education Institutes, College of Life Science, Jinan University, Guangzhou 510632, China; ^b^ School of Energy and Environment, City University of Hong Kong, Tat Chee Avenue, Kowloon, Hong Kong, China; ^c^ Department of Biotechnology, Bharathidasan University, Tiruchirappalli 620024, India.

**
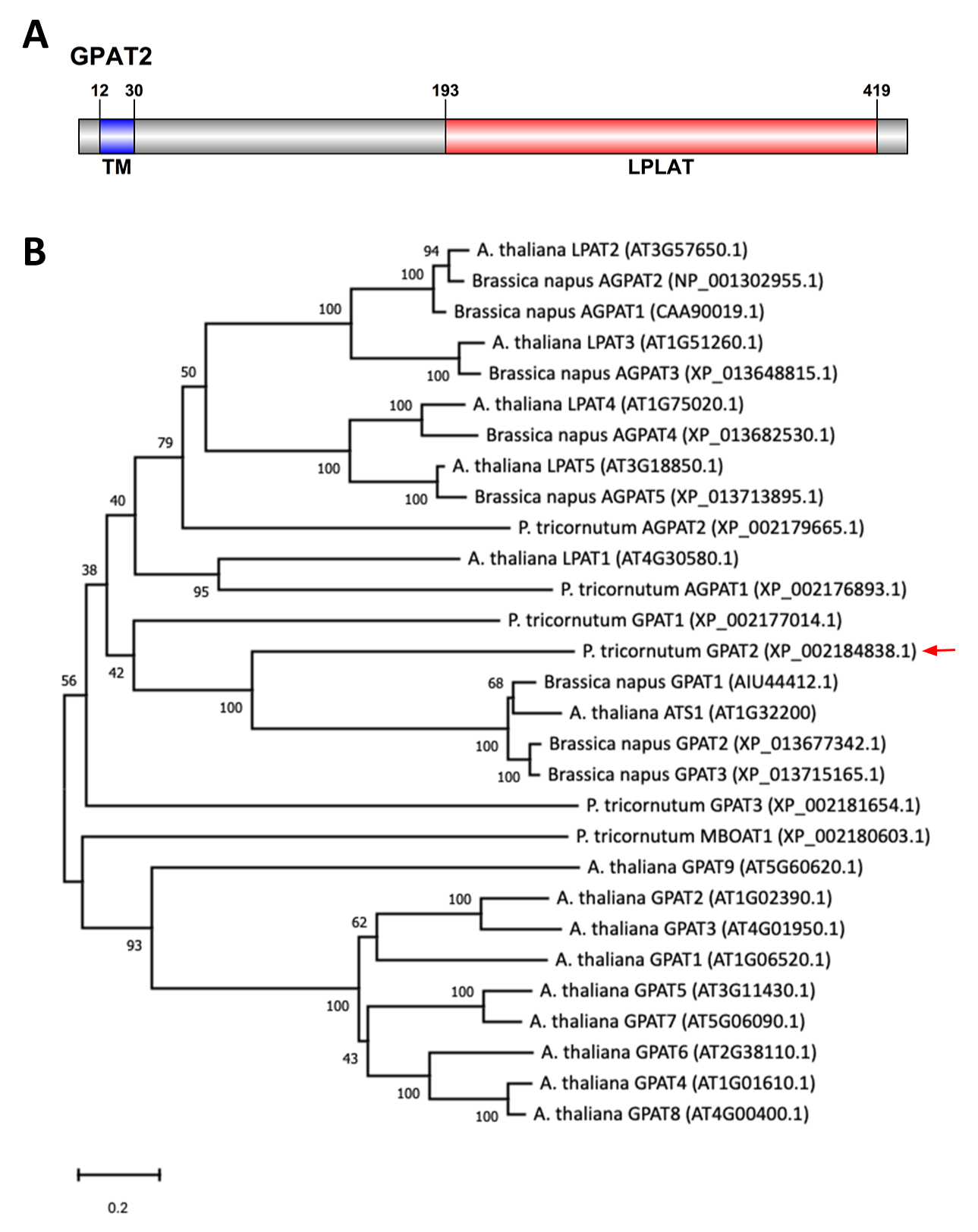
**

**Fig. S1.** *In silico* analyses of GPAT2 sequence. **(A)** Protein structure of GPAT2. **(B)** Phylogeny analysis of GPAT2 from the deduced amino acid sequence of GPAT2 and GPAT of various organisms. Phylogenetic tree was constructed by MEGA using Neighbor joining method. Percentage of the replicate trees where the taxa clustered together in the bootstrap value (500 replicates) are provided in the branches. Arrow mark denotes the GPAT2 of *P. tricornutum*.


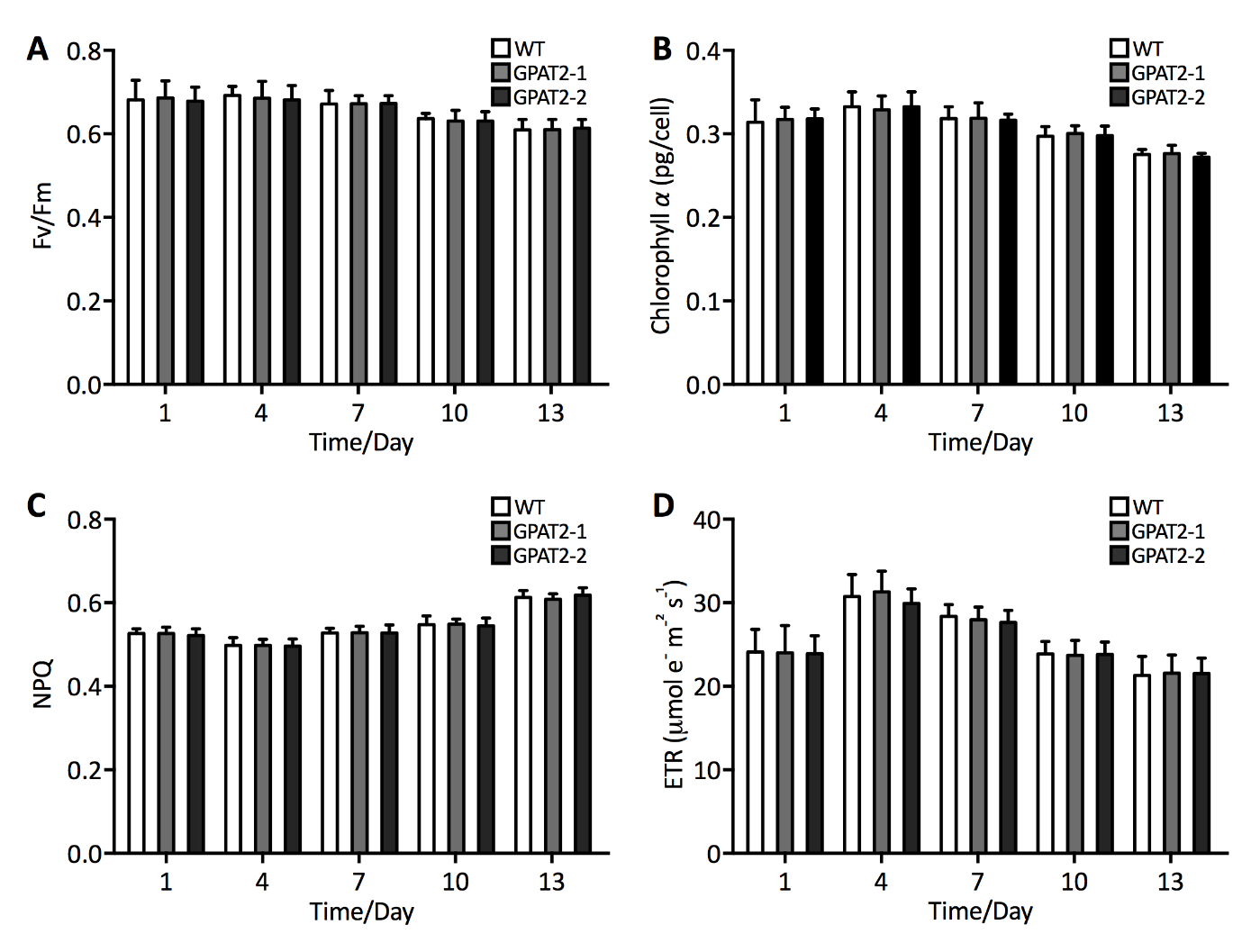


**Fig. S2.** Analyses of photosynthetic parameters in transgenics. **(A)** Maximum quantum yield of photosystem II as indicated by Fv/Fm. **(B)** Determination of chlorophyll α content (pg/cell).

**(C)** Non-photo chemical quenching (NPQ). **(D)** Electron transport rate (ETR) (µmol e^-^m^-2^s^-1^).

Significant difference between WT and transgenics is indicated at the *p* < 0.05 (*) or *p* < 0.01 (**) level. Each value represents the mean ± SD (*n* = 3).


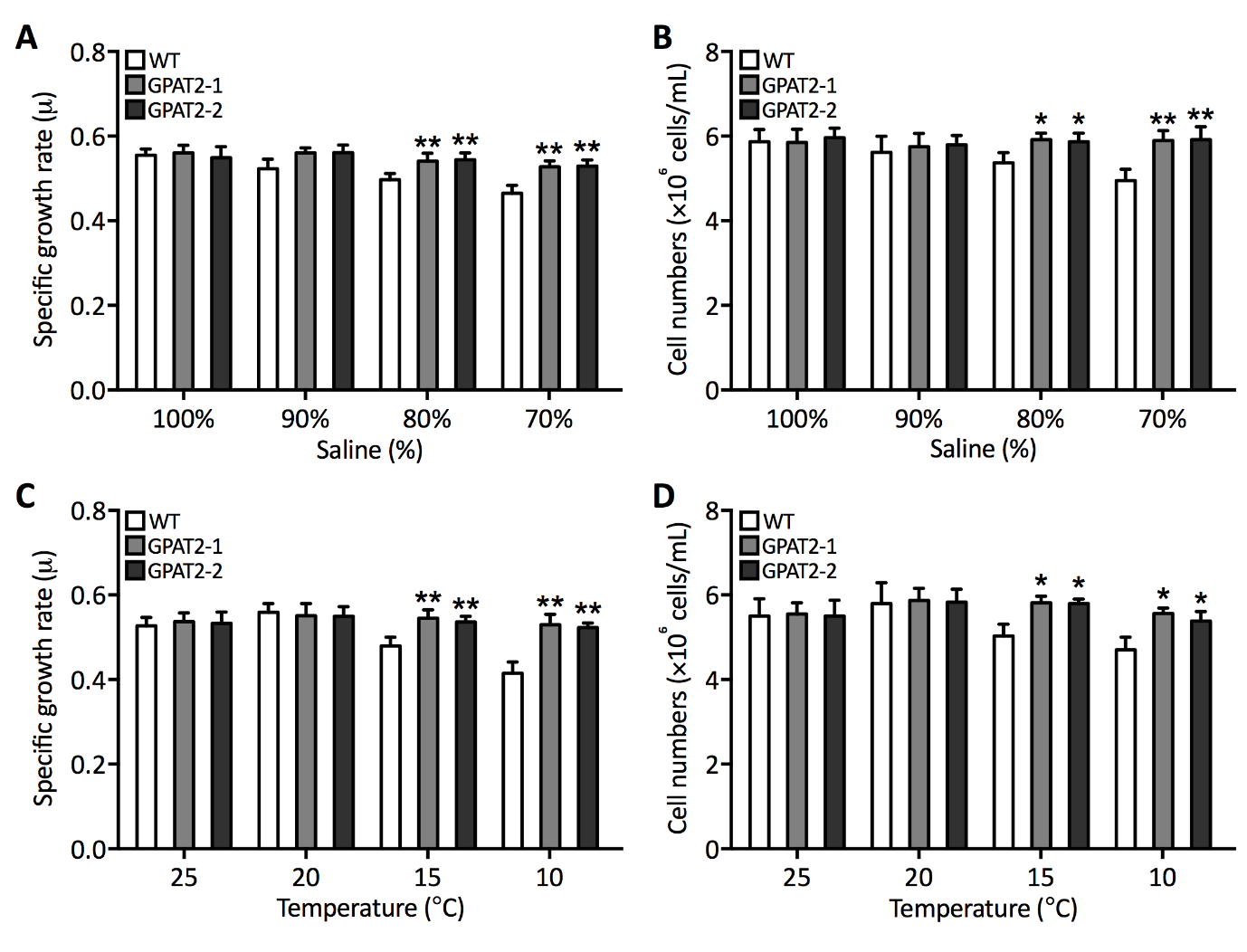


**Fig. S3.** Characterization of physiological parameters under hyposaline and chilling conditions.

Specific growth rate **(A)** and growth curve **(B)** under hyposaline conditions. Specific growth rate **(C)** and growth curve **(D)** at chilling condition (10ºC). Significant difference between WT and transgenics is indicated at the *p* < 0.05 (*) or *p* < 0.01 (**) level. Each value represents the mean ± SD (*n* = 3).


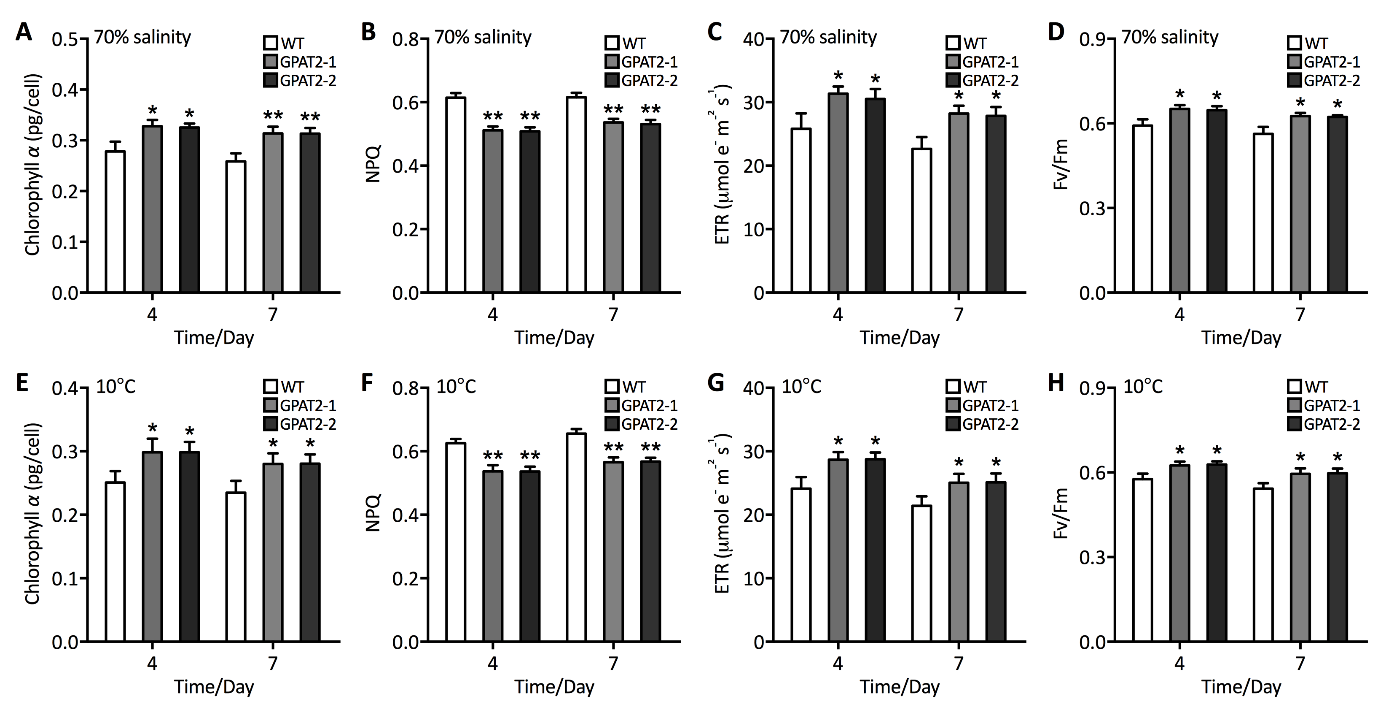


**Fig. S4.** Characterization of photosynthetic parameters under 70 % hyposaline and 10ºC. Determination of **(A)** Chlorophyll α content, **(B)** NPQ, **(C)** ETR (µmol e^-^m^-2^s^-1^) and **(D)** Fv/Fm under 70% hyposaline conditions. Determination of **(E)** Chlorophyll α content, **(F)** NPQ, **(G)** ETR (µmol e^-^m^-2^s^-1^) and **(H)** Fv/Fm at 10ºC. Significant difference between WT and transgenics is indicated at the *p* < 0.05 (*) or *p* < 0.01 (**) level. Each value represents the mean ± SD (*n* = 3).


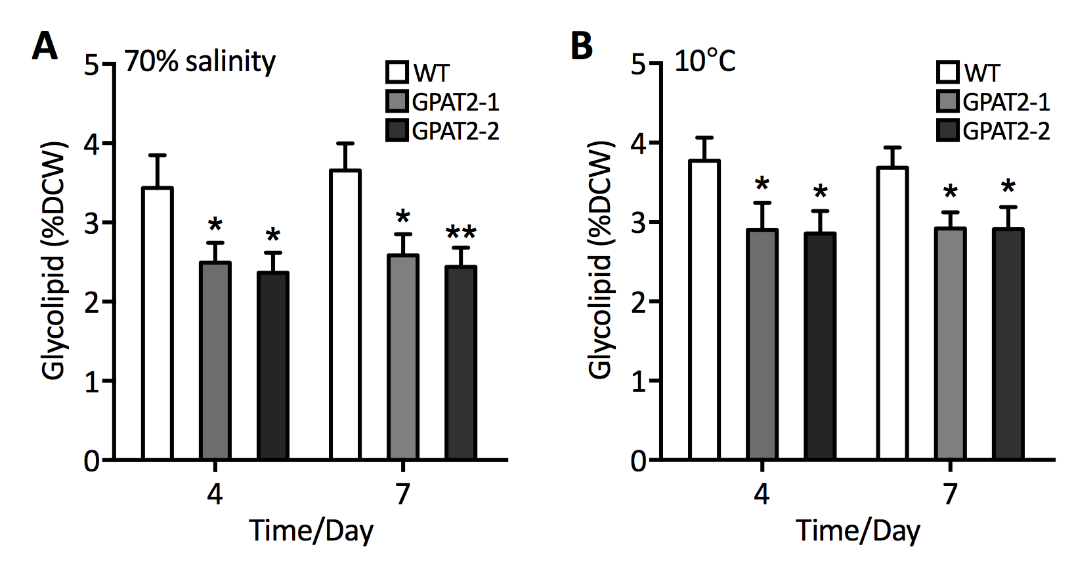


**Fig. S5.** **Analysis of glycolipids in transgenic cells under hyposaline and chilling stress conditions. (A)** Glycolipid content (% DCW) under 70% hyposaline conditions. **(B)** Glycolipid content (% DCW) under chilling condition (10ºC). Significant difference between WT and transgenics is indicated at the *p* < 0.05 (*) or *p* < 0.01 (**) level. Each value represents the mean ± SD (*n* = 3).

**Table S1.** Proportion of C16 and C18 fatty acids in TAG at *sn*-1/3 position.

| Fatty acid | WT | GPAT2-1 | GPAT2-2 |
| --- | --- | --- | --- |
| C16:0 | 27.76 | 48.91 | 48.87 |
| C18:0 | 16.17 | 2.19 | 1.95 |
| C16:1 | 10.55 | 11.94 | 11.62 |
| C18:1 | 14.33 | 2.13 | 1.92 |
| C18:2 | 4.00 | 0.57 | 0.59 |
| C18:3 | 2.94 | 0.41 | 0.93 |

Mean values (*n*=3) are expressed as percentage of each fatty acid composition.

**Table S2.** Proportion of C16 and C18 fatty acids in TAG at *sn*-2 position.

| Fatty acid | WT | GPAT2-1 | GPAT2-2 |
| --- | --- | --- | --- |
| C16:0 | 30.25 | 42.24 | 42.29 |
| C18:0 | 11.95 | 8.21 | 8.30 |
| C16:1 | 12.92 | 9.67 | 9.60 |
| C18:1 | 8.77 | 4.53 | 4.45 |
| C18:2 | 1.72 | 1.00 | 0.93 |
| C18:3 | 1.81 | 0.91 | 0.78 |

Mean values (*n*=3) are expressed as percentage of each fatty acid composition.

**Table S3.** Primers used in this study.

| Gene name | Accession No. | Primer name | Sequence (5’→3’) |
| --- | --- | --- | --- |
| GPAT2 | XP_002181654.1 | GPAT2-f | ACAATTACAATCCAGTGGTACCATGGCGATGCGAGCCGTCA |
|  |  | GPAT2-r | GAGTTTTTGTTCCAGGTGTTGGGTTTGTTTGGATCCATTCCTCC |
|  |  | q-GPAT2-f | AAGACTACCAAGTGAATG |
|  |  | q-GPAT2-r | TGAACTATATGATACGGAAG |
| GPAT1 | XP_002177014.1 | q-GPAT1-f | ACGACAAGGTCGGAACAAAC |
|  |  | q-GPAT1-r | TAAAGGCACCGTCCTTGAAC |
| GPAT3 | XP_002184838.1 | q-GPAT3-f | GCACCCTTTGACTCTAAA |
|  |  | q-GPAT3-r | GAGATCGTAACTGACCAT |
| LPAT1 | XP_002176893.1 | q-LPAT1-f | TACCGATATGATGGAGATGG |
|  |  | q-LPAT1-r | AGACTACCTTATTACCTTGGG |
| LPAT2 | XP_002179665.1 | q-LPAT2-f | CTTCCACCTTCTATTGAG |
|  |  | q-LPAT2-r | TTGATTCGGATGTGTATT |
| LPAT3 | XP_002179875.1 | q-LPAT3-f | CAATTCCTGGATGGATATAC |
|  |  | q-LPAT3-r | GGACCTTACTGAGTTCTT |
| DGAT2A | XP_002184226.1 | q-DGAT2A-f | GATCTGGCCTAAATCCGTCA |
|  |  | q-DGAT2A-r | CGACGATGAGACGATCAAGA |
| DGAT2D | XP_002177637.1 | q-DGAT2D-f | CCACTGTGCTGGGGAAGATA |
|  |  | q-DGAT2D-r | GCAGATGAGCCTTGTCAACC |
| G6PD | XP_002183714.1 | q-G6PD-f | TGACCGCTACGGCATCATAC |
|  |  | q-G6PD-r | GCACATTCCTCCACGTCTCA |
| ME | XP_002180331.1 | q-ME-f | TATGAATGGACCGATGGGCG |
|  |  | q-ME-r | TACATGCAACCGACGTCCAA |
| ACT | XP_002183424.1 | q-ACT-f | AGGCAAAGCGTGGTGTTCTTA |
|  |  | q-ACT-r | TCTGGGGAGCCTCAGTCAATA |
